# Correlation between basic activities of daily living and physical activity in Chinese elderly population with hypertension

**DOI:** 10.1093/cz/zoag013

**Published:** 2026-03-02

**Authors:** Fang Chen, Yiqun Pang, Yi Li, Liming Yan, Xuelin Zhang, Jinxi Zhang, Xinhao Zhuang, Songbo Jia, Weian Tan, Zi Jiao, Bingxiang Xu, Zhixiong Zhou, Qingzhu Yang

**Affiliations:** Sports & Medicine Integrative Innovation Center, Institute of Artificial Intelligence in Sports, Institute of Physical Education and Training, Capital University of Physical Education and Sports, No. 11 North Third Ring West Road, Haidian District, Beijing 100191, China; School of Physical Education, Southwest Petroleum University, No. 8 Xindu Avenue, Xindu District, Chengdu, Sichuan 610500, China; Sports & Medicine Integrative Innovation Center, Institute of Artificial Intelligence in Sports, Institute of Physical Education and Training, Capital University of Physical Education and Sports, No. 11 North Third Ring West Road, Haidian District, Beijing 100191, China; School of Medicine, Tsinghua University, Haidian District, Beijing 100084, China; Sports & Medicine Integrative Innovation Center, Institute of Artificial Intelligence in Sports, Institute of Physical Education and Training, Capital University of Physical Education and Sports, No. 11 North Third Ring West Road, Haidian District, Beijing 100191, China; Sports & Medicine Integrative Innovation Center, Institute of Artificial Intelligence in Sports, Institute of Physical Education and Training, Capital University of Physical Education and Sports, No. 11 North Third Ring West Road, Haidian District, Beijing 100191, China; Sports & Medicine Integrative Innovation Center, Institute of Artificial Intelligence in Sports, Institute of Physical Education and Training, Capital University of Physical Education and Sports, No. 11 North Third Ring West Road, Haidian District, Beijing 100191, China; Sports & Medicine Integrative Innovation Center, Institute of Artificial Intelligence in Sports, Institute of Physical Education and Training, Capital University of Physical Education and Sports, No. 11 North Third Ring West Road, Haidian District, Beijing 100191, China; Sports & Medicine Integrative Innovation Center, Institute of Artificial Intelligence in Sports, Institute of Physical Education and Training, Capital University of Physical Education and Sports, No. 11 North Third Ring West Road, Haidian District, Beijing 100191, China; Sports & Medicine Integrative Innovation Center, Institute of Artificial Intelligence in Sports, Institute of Physical Education and Training, Capital University of Physical Education and Sports, No. 11 North Third Ring West Road, Haidian District, Beijing 100191, China; Key Laboratory of Hebei Province for Molecular Biophysics, School of Health Science & Biomedical Engineering, Hebei University of Technology, No. 5340 Xiping Road, Beichen District, Tianjin 300401, China; Sports & Medicine Integrative Innovation Center, Institute of Artificial Intelligence in Sports, Institute of Physical Education and Training, Capital University of Physical Education and Sports, No. 11 North Third Ring West Road, Haidian District, Beijing 100191, China; Sports & Medicine Integrative Innovation Center, Institute of Artificial Intelligence in Sports, Institute of Physical Education and Training, Capital University of Physical Education and Sports, No. 11 North Third Ring West Road, Haidian District, Beijing 100191, China

**Keywords:** basic activities of daily living, hypertension, physical activity, the elderly

China's rapidly aging population is accompanied by a rising prevalence of chronic diseases, particularly hypertension. In 2015, hypertension affected 34.38% of individuals aged over 45 years and 54.92% of those aged over 60 years. Hypertension contributes to organ failure, cognitive decline, mobility issues, and a decrease in daily living abilities, placing a substantial burden on family caregivers (Hajjar et al. 2011).

Activities of daily living (ADL), which include basic activities of daily living (BADL) and instrumental activities of daily living (IADL), are critical for maintaining independence. BADL comprises essential self-care tasks such as dressing and eating. Notably, while “using a telephone” is traditionally categorized as an IADL, this study incorporated it into the BADL assessment. For older adults with hypertension, the ability to communicate by phone is a fundamental survival skill for emergency response, comparable in necessity to basic self-care. By contrast, IADL encompasses more complex activities requiring decision-making. Mobility impairment in ADL is influenced by gender, age, socioeconomic status, and physical activity (PA), which is a key non-pharmacological strategy for hypertension management and may improve ADL among hypertensive patients (Da 2012).

Most previous studies have emphasized IADL, whereas BADL has received comparatively less attention. This study aims to explore the correlation between BADL and PA in elderly hypertensive patients across China, providing a basis for improving BADL and informing PA and sports-related health policies for hypertensive individuals. Chi-square tests and logistic regression analyses were employed, with detailed methods provided in the Supplementary Materials.

The cross-sectional study involved 3,877 older adults with hypertension from 28 provinces, with women accounting for 52.2% of the sample. Most participants (44.7%) were aged 69–79, 73.5% were married, and 54.2% reported 7–8 hours of sleep daily. The chi-square test revealed a significant association between total metabolic equivalents of PA and BADL impairment (Supplementary Table S2). Correlation analysis showed higher rates of BADL impairment among females (28.0%), those over 79 years (51.2%), widowed individuals (37.3%), and patients with four or more chronic diseases (37.2%) (Supplementary Table S3). Logistic regression identified significant factors influencing BADL, including gender, age, marital status, sleep duration, body mass index (BMI; kg/m²), number of chronic diseases, and PA levels, after applying random oversampling to address class imbalance. These findings provide valuable insights into the factors affecting ADL, with further details in the supplementary materials.

We then modelled determinants of BADL impairment using logistic regression. The dose-response relationship between physical activity volume and BADL impairment is illustrated in Fig. 1. The most powerful factors included: (1) Gender differences: The likelihood of impaired BADL in women was 15% higher than in men. Female hypertensive patients were 1.150 times more likely (OR = 1.150; 95% CI, 1.022–1.294) to experience BADL impairment compared with male patients. (2) Age and BADL impairment: Advancing age was associated with a higher probability of BADL impairment among elderly hypertensive individuals. (3) Marital status: Widowed patients were 1.366 times more likely to have BADL impairment (OR = 1.366; 95% CI, 1.191–1.567) than married patients with surviving spouses. (4) Monthly expenditure: Patients with per capita monthly expenditures of ¥800–¥1,500 and more than ¥1,500 had significantly lower risks of BADL impairment—0.538-fold (OR = 0.538; 95% CI, 0.453–0.640) and 0.789-fold (OR = 0.789; 95% CI, 0.645–0.964), respectively—compared with those spending less than ¥433 per month. (5) Retirement status: Retired participants were 1.246 times more likely (OR = 1.246; 95% CI, 1.051–1.477) to have impaired BADL compared to non-retired individuals. (6) Sleep duration: Participants sleeping 7–8 hours per day had A 36.2% lower risk (OR = 0.638; 95% CI, 0.554–0.735) of BADL impairment compared with those sleeping less than 7 hours per day. (7) BMI: Patients with normal BMI had A 32.8% lower risk (OR = 0.672; 95% CI, 0.497–0.908) of BADL impairment compared with underweight patients. (8) Chronic disease burden: Elderly patients with two chronic diseases were 1.425 times more likely (OR = 1.425; 95% CI, 1.210–1.676) to experience BADL impairment, while those with four or more chronic diseases were 2.432 times more likely (OR = 2.432; 95% CI, 2.035–2.906), compared with patients with hypertension only. (9) PA level: Patients with total PA exceeding 4,224 metabolic equivalent minutes per week (MET-minutes/week) had a significantly reduced risk of BADL impairment (OR = 0.760; 95% CI, 0.642–0.900) compared with those performing less than 1,988 MET-minutes/week.

**Figure 1:**
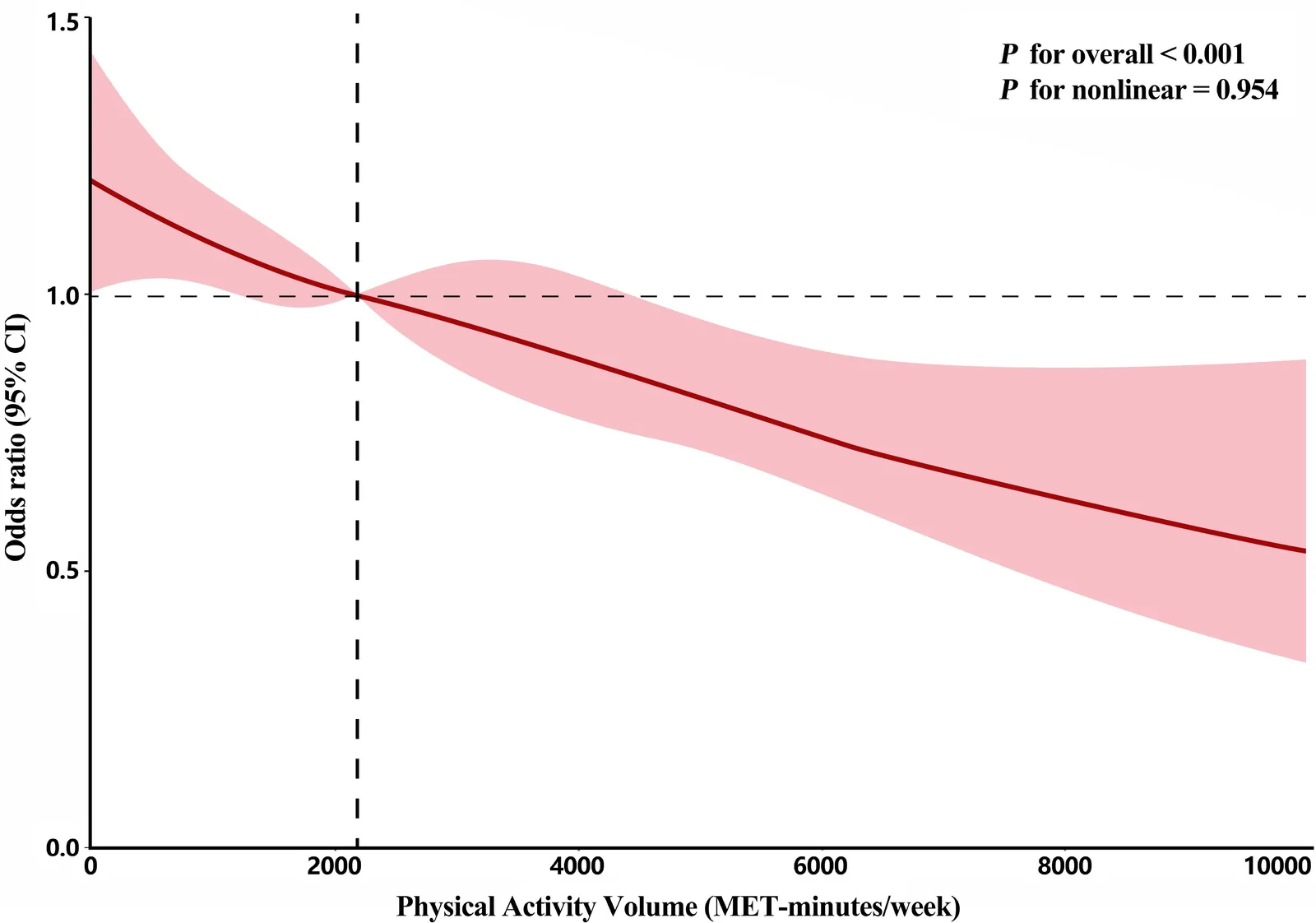
Dose-response curve between PA volume and BADL impairment. Dose-response curve illustrating the association between PA volume (measured in MET-minutes per week) and the odds ratio for limitations in BADL. As PA volume increases, the odds ratio for BADL limitations exhibits a linear decrease. The overall association is statistically significant (P<0.001) with no evident nonlinear relationship (P=0.954).

Collectively, these results highlight key determinants of BADL impairment in older adults with hypertension. Factors such as being female, advanced age, widowed status, retirement, and A higher burden of chronic disease emerged as risk factors. Conversely, higher income levels, sleep duration of 7–8 hours per day, normal BMI, and total PA exceeding 4,224 MET minutes/week were identified as protective factors. These findings underscore the interplay of socioeconomic conditions, health status, and lifestyle behaviors in shaping functional ability among older hypertensive individuals.

As shown in Table 1 (see Supplementary table s4 for the full dataset), PA above 4,224 MET-minutes/week was associated with a lower prevalence of BADL impairment. This aligns with prior evidence and with the Dutch Physical Activity Guidelines, which emphasize that greater volume, duration, and intensity of pa confer greater health benefits, while also discouraging prolonged sedentary behavior (Weggemans et al. 2018). This may be explained by the fact that only sufficient volumes of PA provide benefits such as improving cardiovascular and pulmonary function, enhancing immune function, and effective weight control. Notably, the protective PA thresholds identified in this study (e.g., >1,980 and >4,224 MET-minutes/week) are higher than the WHO recommendation of 600 MET-min/week. This distinction arises because our cut-off points were derived from the statistical quartiles of the study population after random oversampling, specifically to capture the dose-response relationship in this high-risk hypertensive cohort. While the WHO guideline serves as a general baseline, the higher data-driven thresholds observed here reflect the specific activity volumes required to mitigate functional decline in this older demographic. This study also found that moderate sleep duration was associated with reduced BADL impairment, with the lowest impairment observed among those sleeping 7–8 h/day, consistent with evidence that both short and long sleep duration are linked to elevated blood pressure and poorer health outcomes (Czeisler 2015).

**Table 1:** Logistic regression analysis of PA levels and BADL.

Variable	OR	95%Cl
198 to 1,979 MET-minutes/week	1.410^c^	1.192–1.667
1,980 to 4,224 MET-minutes/week	1.082	0.914–1.281
> 4,224 MET-minutes/week	0.760^b^	0.642–0.900
Constant	0.443^c^	—

Note: ^a^, ^b^, ^c^ indicate *P*-values <0.05, *P*-values <0.01, and *P*-values <0.001, respectively.

BADL impairment may reduce PA levels and sleep quality through intertwined physiological and behavioral pathways (Grgic et al. 2018). Physiologically, physical disabilities or neuropathological changes may reduce activity capacity and disrupt circadian rhythms, while behaviorally, discomfort and functional limitations promote avoidance of PA, forming an “impairment–reduced activity–worsened function feedback loop (Liu et al. 2024). Existing statistical approaches, including cross-lagged and multivariate regression models, support predominantly unidirectional relationships among these variables, providing an evidence base for targeted interventions.

Although based on human data, our findings have broader relevance for zoological studies of aging. Across taxa, locomotor activity and daily functional behaviors are closely linked to survival, foraging efficiency, and social participation, and their decline is a hallmark of senescence. The positive association between physical activity and preserved functioning supports life-history models suggesting that sustained movement capacity prolongs behavioral autonomy. Evidence from birds and mammals indicates conserved mechanisms linking activity, muscle function, and functional aging, highlighting physical activity as an integrative trait for cross-species studies of aging and performance-related fitness.

This study has several limitations: the cross-sectional design limits causal inference, self-reported PA may introduce recall bias, potential confounders were not fully controlled, and MET-minute calculations may not perfectly reflect true energy expenditure in older Chinese adults. However, the large nationally representative sample and focused analysis of the PA-BADL relationship strengthen the validity and generalizability of the findings.

## Supplementary Material

zoag013_Supplementary_Data

## Artificial intelligence (AI)-assisted technologies

Assistance is provided by ChatGPT in sentence editing.
