# Supplementary material for: Correlation between basic activities of daily living and physical activity in Chinese elderly population with hypertension
Source: Curr Zool. 2026 Mar 2;72(5):759–61. doi: 10.1093/cz/zoag013 (PMC13690590; doi:10.1093/cz/zoag013)
Supplement: Supplementary file 1 [file zoag013_supplementary_data.zip › Supplementary Materials.docx]

**Supplementary Methods**

**Participants**

This cross-sectional study was conducted based on the China Longitudinal Aging Social Survey (CLASS) survey in 2020. CLASS is conducted by the Renmin University of China which is aimed at developing a basic understanding of the situation of elders and providing a scientific basis for the formulation of a rational and effective policy on ageing. This national baseline survey was conducted in 2020 and covered 28 provinces nationwide. The CLASS questionnaire included demographic characteristics of elderly, physical health status, economic status mental health status, and need for elderly services. It was approved by the Ethics Review Committee of Renmin University of China, but the ethics approval number has not been publicly available. and informed consent was required of all participants.

A total of 11,398 participants were included who were aged over 59 years in 28 provinces across China. Then those who lacked data from blood pressure, physical activity, and BADLs questionnaires were excluded. Next, participants who did not fill in their self-report that they had a chronic condition of hypertension or had not been diagnosed by a physician were excluded, and the final study participants consisted of 3,877 hypertensive patients aged 59 years or older.

**Assessment of physical activity**

In the questionnaire, physical activity is categorized into three levels: vigorous physical activity (VPA), moderate physical activity (MPA), and light physical activity (LPA). VPA involves exertion that requires significant effort, rapid breathing, noticeably increased heart rate, and substantial sweating. MPA requires a moderate effort, slightly faster breathing than usual, increased heart rate, and mild sweating. LPA involves breathing and heart rate during activity that are essentially the same as at rest. During the survey, participants were asked questions related to the following three aspects: (1) In the last 7 days, how many days did you engage in high-intensity physical activity lasting at least 10 minutes per session, such as lifting weights, digging, aerobic exercise, or fast cycling? What was the duration of each day? (2) In the last 7 days, how many days did you engage in moderate-intensity physical activity lasting at least 10 minutes per session, such as lifting light items, cycling at a regular pace, or playing tennis and badminton (excluding walking)? What was the duration of each day? (3) In the last 7 days, how many days did you engage in walking activities lasting at least 10 minutes each time? What was the duration of each day? These walking activities include walking from one place to another at home or walking during leisure and exercise.

The physical activity volume (PAV) can be expressed using the total metabolic equivalent (MET) values, calculated as follows: PAV = 8.0 × weekly duration of VPA + 4.0 × weekly duration of MPA + 3.3 × weekly duration of LPA. Finally, quartiles were applied to categorize the total metabolic equivalent into four classes. Analysis of patients' basic activity capacity revealed a ratio of approximately 3:1 between those unimpaired and those impaired, resulting in a severe imbalance in the positive and negative sample proportions. To prevent machine learning algorithms from favoring the dominant class, which could lead to a decrease in model performance, this study performed Random Oversampling on the original data, adjusting the ratio of affected to unaffected to 1:1. After this adjustment, the total sample size was 5802. Due to changes in the sample distribution, the quartiles of patients' total metabolic equivalents also underwent adjustments. As a result, the reclassified categories for patients' total metabolic equivalents were as follows: below 198 MET-minutes/week, 198 to 1979 MET-minutes/week, 1980 to 4224 MET-minutes/week, and above 4224 MET-minutes/week.

**Assessment of basic activities of daily living**

In the CLASS survey, the scale for basic activities of daily living comprises 11 indicators, namely, making phone calls, grooming, dressing, bathing, eating, taking medication, urination, defecation, using the toilet, bedside mobility, and indoor ambulation. Each item is categorized into three levels: "does not need assistance," "needs some assistance," and "completely unable to do." If the response indicates "completely unable to do" or "needs some assistance," it is classified as impaired; if the response is "does not need assistance," it is classified as unimpaired. If any of the activities in the BADL assessment are impaired, it is considered as BADL impairment.

**Covariates**

Covariates in the present study included (1) general demographic variables, such as gender (male and female), age (59~69, 70~79, and >79), household (rural and urban), marital status (married with a spouse, widowed, divorced, and unmarried), and per capita monthly expenditures (¥0~433, ¥434~800, ¥801~1500, and more than ¥1500), retirement status (retired and non-retired); (2) living habits and physical condition variables, including sleep duration (< 7h/d, 7~8h/d and >8h/d), smoking status (smoking or non-smoking), BMI (thin: BMI< 18.5 kg/m^2^, normal: 18.5 kg/m^2^ ≤ BMI ≤23.9 kg/m^2^, overweight: 24.0 kg/m^2^ ≤ BMI ≤ 27.9 kg/m^2^, and obese: BMI ≥ 28.0 kg/m^2^), number of chronic disease (1, 2, 3 and ≥4), and the number of hospitalization (<5 times or ≥5 times).

**Statistical analysis**

The study patients' general demographic characteristics, lifestyle habits, and physical condition were expressed as frequencies and percentages. The chi-square test was used to study the correlation between BADL and influencing factors such as physical activity. Finally, the Logistic regression analysis model was used to analyze the effect of physical activity on BADL in elderly hypertensive patients under multiple factors. To make the model analysis results more accurate, this study oversampled the data before analysis. IBM SPSS Statistics 27 software was employed for all the above analyses. The test level ɑ= 0. 05.

**Supplementary tables S1-S3**

**Demographic Characteristics**

The demographic characteristics of the cross-sectional study, comprising 3877 elderly hypertensive patients from 28 provinces in China, underwent rigorous statistical analysis to procure essential baseline information. A detailed breakdown is provided in Table S1. Gender distribution revealed 1,852 (47.8%) males and 2,025 (52.2%) females. The majority of patients (44.7%) fell within the 69-79 years age bracket, and 53.3% were of rural household registration. Regarding marital status, the majority of patients (73.5%) were married with a spouse. The number of patients with monthly expenditure of less than ¥450, ¥450-¥800 and ¥800-¥1,500 had little difference, accounting for 25.3%, 26.2% and 26.3% respectively. Retirement status indicated that 54.9% of patients were not retired. Analysis of lifestyle habits encompassed sleep duration and smoking status. Notably, 54.2% of patients reported a daily sleep duration between 7 and 8 hours, while 28.8% were smokers. Patients' physical condition was multifaceted, including Body Mass Index (BMI), number of chronic diseases, and the number of hospitalizations. The majority of patients had normal weight (60.9%), followed by those classified as overweight (30.6%), with the fewest patients having a thin body (3.7%). In terms of chronic diseases, 31.7% of patients suffered from two. The number of Hospitalization revealed that 99.7% of patients had experienced fewer than five hospitalizations in the previous year.

Table S1. Demographic characteristics of patients

| Characteristics | Overall Sample (n = 3877) | |
| --- | --- | --- |
|  | n | % |
| Gender |  |  |
| Male | 1852 | 47.8 |
| Female | 2025 | 52.2 |
| Age |  |  |
| 59-69 | 1489 | 38.4 |
| 69-79 | 1734 | 44.7 |
| >79 | 654 | 16.9 |
| Household registration |  |  |
| Rural | 2067 | 53.3 |
| Urban | 1810 | 46.7 |
| Marital status |  |  |
| Married | 2850 | 73.5 |
| Widowed | 982 | 25.3 |
| Divorce | 26 | 0.7 |
| Unmarried | 19 | 0.5 |
| Per capita monthly expenditures |  |  |
| <¥450 | 982 | 25.3 |
| ¥450-¥800 | 1017 | 26.2 |
| ¥800-¥1,500 | 1020 | 26.3 |
| >¥1500 | 858 | 22.1 |
| Retirement status |  |  |
| Yes | 1747 | 45.1 |
| No | 2130 | 54.9 |
| Sleep duration |  |  |
| <7h/d | 898 | 23.2 |
| 7-8h/d | 2103 | 54.2 |
| >8h/d | 876 | 22.6 |
| Smoking status |  |  |
| Yes | 1118 | 28.8 |
| No | 2759 | 71.2 |
| BMI |  |  |
| Thin | 142 | 3.7 |
| Normal weight | 2361 | 60.9 |
| Overweight | 1187 | 30.6 |
| Obese | 187 | 4.8 |
| Number of chronic diseases |  |  |
| One chronic disease | 940 | 24.2 |
| Two chronic diseases | 1230 | 31.7 |
| Three chronic diseases | 912 | 23.5 |
| Four or more chronic diseases | 795 | 20.5 |
| Hospitalizations |  |  |
| <5 times | 3866 | 99.7 |
| ≥5 times | 11 | 0.3 |

**Correlation Analysis Between physical activity volume and BADL**

To investigate the relationship between patients' PA and BADL, a chi-square test was conducted. The results, as presented in Table S2, demonstrate a significant association between the total metabolic equivalents of PA and the prevalence of impaired BADL (*P* < 0.001). Notably, the number of patients with impaired BADL was consistently lower compared to those with unimpaired BADL across different ranges of total metabolic equivalents of PA. In addition, when the physical activity metabolic equivalent exceeded 264 MET-minutes /week, the impaired rate of BADL decreased significantly with the increase of the total metabolic equivalent of PA. (*P* < 0.001).

Table S2. Correlation analysis between PA levels and BADL in patients

| Variable | n | BADL | | χ^2^ |
| --- | --- | --- | --- | --- |
|  |  | Ability | Disability |  |
| TPA |  |  |  | 57.118*** |
| <198 MET-minutes/week | 979 | 694(70.90%) | 285(29.10%) |  |
| 198 to 1979 MET-minutes/week | 960 | 664(69.20%) | 296(30.80%) |  |
| 1980 to 4224 MET-minutes/week | 987 | 757(76.70%) | 230(23.30%) |  |
| > 4224 MET-minutes/week | 951 | 786(82.60%) | 165(17.40%) |  |
| Total | 3877 | 2901(74.80%) | 976(25.20%) |  |

note: *, **, *** indicate *P*-values <0.05, *P*-values <0.01, and *P*-values <0.001, respectively.

**Correlation Analysis Between Other Factors and BADL**

In this study, a comprehensive correlation analysis was undertaken to explore the connection between various covariates (including gender, age, household registration, marital status, per capita monthly expenditure, retirement status, sleep duration, smoking status, BMI, number of chronic diseases, and the number of hospitalization) and the BADL among hypertensive patients. The detailed results of this analysis are summarized in Table S3. The prevalence of impaired BADL in female hypertensive patients reached 28%, exceeding that observed in male patients (22.1%). Moreover, patients aged over 79 exhibited the highest prevalence of impaired BADL, approximately four times higher than those aged 59-69, reaching 51.2%. This pattern suggests a proportional increase in BADL impairment with advancing age. Urban hypertensive patients displayed a higher BADL impairment rate (27.4%) compared to their rural counterparts. Among marital status categories, widowed patients registered the highest BADL impairment rate (37.3%), which was twice that of divorced and unmarried patients. Patients with a per capita monthly expenditure exceeding 1,500 yuan demonstrated the highest BADL impairment rate at 31.5%. Additionally, retired patients exhibited a higher BADL impairment rate (27.6%) compared to those who were not retired. Overall, the findings underscore the significant impact of gender, age, household registration, marital status, per capita monthly expenditure, retirement status, sleep duration, BMI, and number of chronic disease on patients' BADL (P < 0.05).

Table S3. Correlation analysis between other factors and BADL in patients

| Variable | n | BADL | | χ2 |
| --- | --- | --- | --- | --- |
|  |  | Ability | Disability |  |
| Gender |  |  |  |  |
| Male | 1852 | 1442(77.9%) | 410(22.1%) | 17.349*** |
| Female | 2025 | 1459(72.0%) | 566(28.0%) |  |
| Age |  |  |  |  |
| 59-69 | 1489 | 1299(87.2%) | 190(12.8%) | 358.048*** |
| 69-79 | 1734 | 1283(74.0%) | 451(26.0%) |  |
| >79 | 654 | 319(48.8%) | 335(51.2%) |  |
| Household registration |  |  |  |  |
| Rural | 2067 | 1587(76.8%) | 480(23.2%) | 8.956** |
| Urban | 1810 | 1314(72.6%) | 496(27.4%) |  |
| Marital status |  |  |  |  |
| Married | 2850 | 2247(78.8%) | 603(21.2%) | 102.902*** |
| Widowed | 982 | 616(62.7% | 366(37.3%) |  |
| Divorce | 26 | 22(84.6%) | 4(15.4%) |  |
| Unmarried | 19 | 16(84.2%) | 3(15.8%) |  |
| Per capita monthly expenditures |  |  |  |  |
| Quarter 1 | 982 | 712(72.5%) | 270(27.5%) |  |
| Quarter 2 | 1017 | 800(78.7%) | 217(21.3%) | 36.230*** |
| Quarter 3 | 1020 | 801(78.5%) | 219(21.5%) |  |
| Quarter 4 | 858 | 588(68.5%) | 270(31.5%) |  |
| Retirement status |  |  |  |  |
| No | 2130 | 1636(76.8%) | 494(23.2%) | 9.854** |
| Yes | 1747 | 1265(72.4%) | 482(27.6%) |  |
| Sleep duration |  |  |  |  |
| <7h/d | 898 | 623(69.4%) | 275(30.6%) | 55.667*** |
| 7-8h/d | 2103 | 1674(79.6%) | 429(20.4%) |  |
| >8h/d | 876 | 604(68.9%) | 272(31.1%) |  |
| Smoking status |  |  |  |  |
| No | 2759 | 2059(74.6%) | 700(25.4%) | 0.198 |
| Yes | 1118 | 842(75.3%) | 276(24.7%) |  |
| BMI |  |  |  |  |
| Thin | 142 | 97(68.3%) | 45(31.7%) | 30.439*** |
| Normal weight | 2361 | 1826(77.3%) | 535(22.7%) |  |
| Overweight | 1187 | 862(72.6%) | 325(27.4%) |  |
| Obese | 187 | 116(62.0%) | 71(38.0%) |  |
| Number of chronic diseases |  |  |  |  |
| One chronic disease | 940 | 797(84.8%) | 143(15.2%) | 118.489*** |
| Two chronic diseases | 1230 | 949(77.2%) | 281(22.8%) |  |
| Three chronic diseases | 912 | 656(71.9%) | 256(28.1%) |  |
| Four or more chronic diseases | 795 | 499(62.8%) | 296(37.2%) |  |
| Hospitalizations |  |  |  |  |
| <Five times | 3866 | 2894(74.9%) | 972(25.1%) | 0.733 |
| ≥Five times | 11 | 7(63.6%) | 4(36.4%) |  |

note: *, **, *** indicate *P*-values <0.05, *P*-values <0.01, and *P*-values <0.001, respectively.

**Logistic regression analysis of PA levels and BADL**

This cross-sectional study investigated the current status of basic BADLs in Chinese elderly hypertensive patients and analyzed the correlation between PA and basic BADLs. Besides, it explored the effects of gender, age, marital status, economic status, place of residence, and number of chronic diseases on BADLs of elderly hypertensive patients. The major findings of this study are as follows: 1) The total rate of BADL impairment in Chinese elderly hypertensive patients was 25.2%, and among the 11 activities regarding BADL, the highest impaired rate was the ability to make phone calls, which was 13%. 2) Among the various influencing factors, females, higher age, widowed, retired and a high number of chronic diseases are the risk factors for impaired BADL in elderly hypertensive patients; whereas, high income, sleep 7-8 h/d, normal BMI, and total metabolic equivalents above 4,224 MET-minutes/week or more are protective factors for patients with BADL impairment.

Table S4. Logistic regression model

| Variable | OR | 95%Cl |
| --- | --- | --- |
| Gender (Male) | Reference |  |
| Female | 1.150* | 1.022-1.294 |
| Age (59-69) | Reference |  |
| 69-79 | 2.332*** | 2.042-2.664 |
| >79 | 6.376*** | 5.323-7.639 |
| Household registration (Rural) | Reference |  |
| Urban | 0.945 | 0.797-1.120 |
| Marital status (Married) | Reference |  |
| Widowed | 1.366*** | 1.191-1.567 |
| Divorce | 0.626 | 0.274-1.432 |
| Unmarried | 0.607 | 0.230-1.604 |
| Per capita monthly expenditures (Quarter 1) | Reference |  |
| Quarter 2 | 0.679*** | 0.579-0.797 |
| Quarter 3 | 0.538*** | 0.453-0.640 |
| Quarter 4 | 0.789* | 0.645-0.964 |
| Retirement status (No) | Reference |  |
| Yes | 1.246* | 1.051-1.477 |
| Sleep duration (＜7h/d) | Reference |  |
| 7-8h/d | 0.638*** | 0.554-0.735 |
| >8h/d | 1.139 | 0.964-1.345 |
| BMI (Thin) | Reference |  |
| Normal weight | 0.672* | 0.497-0.908 |
| Overweight | 0.980 | 0.718-1.338 |
| Obese | 1.247 | 0.844-1.842 |
| Number of chronic diseases (One chronic disease) | Reference |  |
| Two chronic diseases | 1.425*** | 1.210-1.676 |
| Three chronic diseases | 1.970*** | 1.661-2.336 |
| Four or more chronic diseases | 2.432*** | 2.035-2.906 |
| PA (<198 MET-minutes/week) | Reference |  |
| 198 to 1979 MET-minutes/week | 1.410*** | 1.192-1.667 |
| 1980 to 4224 MET-minutes/week | 1.082 | 0.914-1.281 |
| > 4224 MET-minutes/week | 0.760** | 0.642-0.900 |
| Constant | 0.443*** |  |

note: *, **, *** indicate *P*-values <0.05, *P*-values <0.01, and *P*-values <0.001, respectively.

**Supplementary figures S1**

**Receiver Operating Characteristic curve**

Figure S1 The ROC curve of the logistic regression model. After fitting the logistic regression model, we tested the fitness of the model, and the final area under the curve (AUC) was 0.74 (Figure 1). We believed that the model had fitting ability.
